# Supplementary material for: Wet Bulb Globe Temperature and Recorded Occupational Injury Rates among Sugarcane Harvesters in Southwest Guatemala
Source: Int J Environ Res Public Health. 2020 Nov 6;17(21):8195. doi: 10.3390/ijerph17218195 (PMC7664243; doi:10.3390/ijerph17218195)
Supplement: Supplementary file 1 [file ijerph-17-08195-s001.zip › Resubmission STables.docx]

**Table S1.** Estimated Risk Ratios (RR) for recorded occupational injuries among sugarcane harvesters in Southwestern Guatemala – 2014-2018. All models adjusted for total number of workers and harvest season.

|  | **All injuries^1^** | |
| --- | --- | --- |
|  | **RR (95% CI)** | **p-value** |
| Centered^2^ WBGT_max_ | 1.03 (0.94, 1.14) | 0.381 |
| Centered WBGT_max_^2^ | 1.04 (1.00, 1.08) | 0.149 |
| Average daily tons cut | 1.09 (0.89, 1.32) | 0.372 |
| Acclimatization period^3^ | 1.17 (0.64, 1.99) | 0.701 |

^1^All recorded occupational injuries to include: falls, hit by a falling object, slips, caught or stuck between an object, strains or sprains, exposure to extreme heat (non-ambient such as steam), exposure to electrical current, exposure to a harmful substance or radiation, chemical accidents, cut with an agricultural tool, vehicular accidents, bites from snakes or insects, agricultural incidents, or other.

^2^WBGT_max_ was centered on 34°C

^4^Acclimitization period occurred during the first 2 weeks of every hearvest.

**Table S2.** Estimated Risk Ratios (RR) for recorded occupational injuries^1^ among sugarcane harvesters in Southwestern Guatemala for the 2015-2016 harvest season. All models adjusted for total number of workers.

|  | **El Balasamo** | | **Cengicaña** | |
| --- | --- | --- | --- | --- |
|  | **RR (95% CI)** | **p-value** | **RR (95% CI)** | **p-value** |
| Centered^2^ WBGT_mean_ | 1.10 (0.92, 1.32) | 0.291 | 1.01 (0.70, 1.40) | 0.964 |
| Centered WBGT_mean_^2^ | 1.01 (0.90, 1.10) | 0.922 | 0.91 (0.76, 1.06) | 0.256 |
| Average daily tons cut | 1.04 (0.75, 1.41) | 0.831 | 1.02 (0.74, 1.39) | 0.823 |
| Acclimatization period^3^ | 0.48 (0.11, 1.41) | 0.240 | 0.49 (0.11, 1.44) | 0.252 |

^1^All recorded occupational injuries to include: falls, hit by a falling object, slips, caught or stuck between an object, strains or sprains, exposure to extreme heat (non-ambient such as steam), exposure to electrical current, exposure to a harmful substance or radiation, chemical accidents, cut with an agricultural tool, vehicular accidents, bites from snakes or insects, agricultural incidents, or other.

^2^WBGT_mean_ was centered on 30°C

^4^Acclimitization period occurred during the first 2 weeks of every harvest.
