# Supplementary figures and images for: Wet Bulb Globe Temperature and Recorded Occupational Injury Rates among Sugarcane Harvesters in Southwest Guatemala
Source: Int J Environ Res Public Health. 2020 Nov 6;17(21):8195. doi: 10.3390/ijerph17218195 (PMC7664243; doi:10.3390/ijerph17218195)

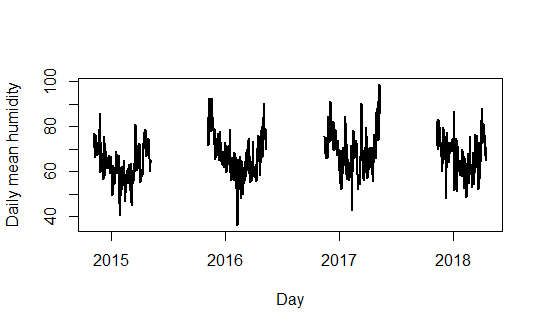

Supplement: Supplementary file 1 [file ijerph-17-08195-s001.zip › Figure S1.tiff]

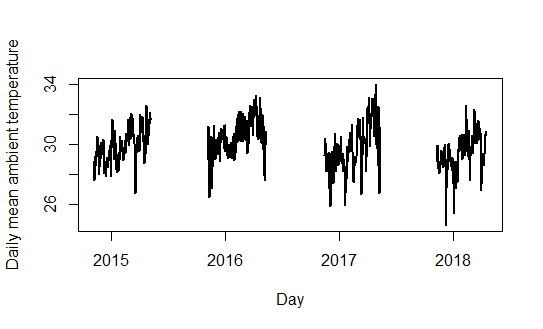

Supplement: Supplementary file 1 [file ijerph-17-08195-s001.zip › Figure S2.tiff]

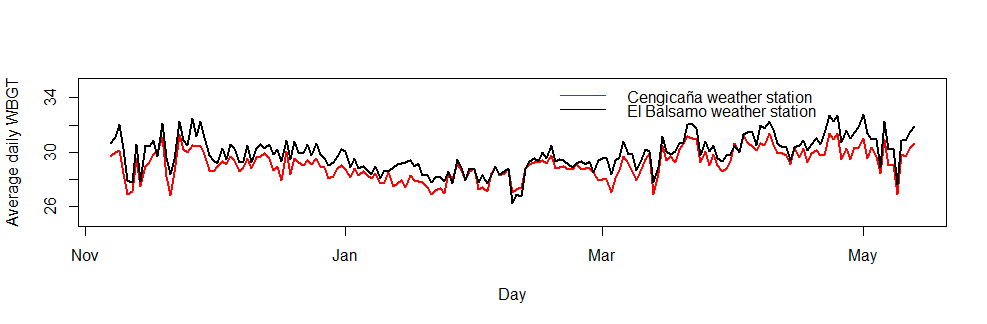

Supplement: Supplementary file 1 [file ijerph-17-08195-s001.zip › Figure S3.tiff]
